# Salt Concentration Control of Polysulfide Dissolution, Diffusion, and Reactions in Lithium–Sulfur Battery Electrolytes

**DOI:** 10.1021/acsaem.5c02378

**Published:** 2025-10-31

**Authors:** N. Tan Luong, Aginmariya Kottarathil, Władysław Wieczorek, Patrik Johansson

**Affiliations:** † Department of Physics, 11248 Chalmers University of Technology, 412 96 Gothenburg, Sweden; ‡ Faculty of Chemistry, 49566 Warsaw University of Technology, 00664 Warsaw, Poland; § ALISTORE—European Research Institute, CNRS FR 3104, Hub de l’Energie, 15 Rue Baudelocque, 80039 Amiens, France

**Keywords:** Li−S, battery, electrolytes, polysulfides, *operando* Raman spectroscopy, DFT

## Abstract

Lithium–sulfur (Li–S) batteries suffer from the dissolution of sulfur and polysulfide (PS) species in the electrolyte, leading to capacity loss, instability, and a shortened lifespan. While highly concentrated electrolytes have been explored to address this issue, the underlying mechanisms of S/PS dissolution and subsequent diffusion, particularly concerning the specific behavior of long- and short-chain PSs under varying states of charge (SOC), remain poorly understood. We here employ *operando* Raman spectroscopy to semiquantitatively monitor PS solubility and migration across a wide range of LiTFSI concentrations in DME:DOL (1:1, v/v). We find that both PS dianions (S_4–8_^2–^) and trisulfur radicals (S_3_^•–^) decrease at the lithium anode with increasing electrolyte salt concentration (0.3–7.0 m), indicating reduced solubility and slower transport. Notably, the concentration of S_3_^•–^ decreases more rapidly than that of its parent PS S_6_^2–^, suggesting less favorable radical formation pathways in highly concentrated electrolytes, potentially due to Li–TFSI–PS adduct formation. These changes result from shifts in the local solvation structure at high salt concentration, thereby controlling the solubility, transport, and chemical pathways of polysulfides in the electrolyte. By providing the real-time dynamics of long- and short-chain PSs, this work advances the mechanistic understanding of PSs in order to provide valuable insight for further improvement of Li–S battery performance.

## Graphical abstract

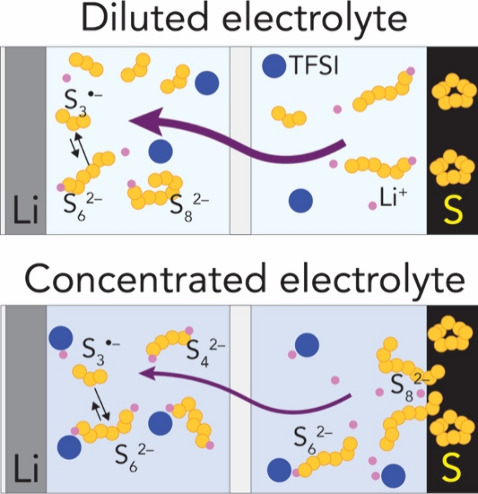

## Introduction

Polysulfides (PSs) are crucial intermediates in the operation of Li–S batteries, a technology possibly capable of delivering twice the specific energy of conventional lithium-ion batteries.^1^ These species, with the general formula Li_*x*_S_*n*_ (*x* = 0–2, *n* = 2–8), are generated through complicated and not completely identified and understood sulfur redox conversion mechanisms.^2–4^ PSs are inherently soluble in liquid electrolytes, with the solubility scaling with the S–S chain length,^5^ allowing them to diffuse to and react with the lithium metal anode (PS shuttling mechanism) during cycling,^6,7^ eventually causing a loss of capacity and shortened lifespan.^4,6,8,9^ Extensive research on electrolyte formulations^8,10–20^ has collectively improved Li–S battery cycling performance^12,13^ and specific energy density,^14^ albeit still at the lab-scale level. Persistent challenges of the Li–S battery technology stem from an incomprehensive understanding of how the electrolyte formulation controls PS dissolution, transport, and reactivity.

The standard Li–S battery electrolyte is 1.0 molar (M) lithium bis­(trifluoromethanesulfonyl)­imide (LiTFSI) in an equal volume mixture of 1,2-dimethoxyethane (DME) and 1,3-dioxolane (DOL). It dissolves considerable amounts of PSs, leading to severe PS shuttling and hence poor stability.^5^ Electrolytes with the same salt concentration but better solvating solvents dissolve even more PSs.^5^ They are, however, often corrosive to the lithium metal anode^15^ but do effectively utilize sulfur redox conversion by notably stabilizing S_3_^•–^ and S_6_^2–^.^15,16,21^ Salt concentrations lower than 1.0 M can be applied, but then alongside solvents with less solvating power toward PSs, such as fluorinated ethers.^17^ On the other hand, increasing the salt concentration effectively decreases the PS solubility, and thus, performance is (hopefully/possibly) improved.^10,12,18–20^ The latter is, however, still in doubt due to intrinsic drawbacks like higher viscosity and reduced ionic conductivity^12^ that can lead to cell failure from high polarization. Lower PS solubility under salt-rich conditions is thermodynamically explained by the common ion effect of the abundant Li^+^ concentration^10^ and by the few free solvent molecules available.^10,12,18^ The higher viscosity also slows PS diffusion.

At the molecular level, strong coordination of the solvent with Li^+^ renders solvent-separated ion pairs (SSIPs) and thus amplifies the complete dissolution and dissociation of PSs into mono- (S_*n*_^–^) and dianions (S_*n*_^2–^), as well as radical anions (S_*n*_^•–^). However, as solvation strength is shared and regulated with the presence of salt in the electrolyte, it may facilitate the formation of contact ion pairs (CIPs) and/or larger aggregates (AGGs), as for any type of salt in high ionic strength media.^22,23^ Indeed, several studies have argued the existence of CIPs and AGGs of PSs due to ionic association.^24–29^ Furthermore, solvent molecules can be replaced by salt anions to form anion-solvated PSs,^30^ especially at salt concentrations that promote Li^+^–anion interactions over Li^+^–solvent interactions. These anion-solvated PSs could facilitate different sulfur reduction pathways,^30^ possibly explaining the shifts in PS chain-length speciation at high salt concentrations,^18^ but these changes may also result from the complicated chemical equilibria. It is, therefore, challenging to assess these dynamics at the same time with the coexistence of multiple PS chain lengths, whose concentrations continuously change across different cell states of charge (SOC). As a result, *ex situ* assessments of the physical properties and local electrolyte structure most often fail to accurately capture the dynamic solvation, transport, and reactivity of PSs. *In situ*/*operando* spectroscopy,^21,31–35^ diffraction,^2,3^ and imaging,^18,36^ on the other hand, have recently revealed solvent-dependent PS dissolution, diffusion, and conversion mechanisms in Li–S batteries and may therefore also uncover how these processes vary with salt concentration.

Here, we aim to offer new insights by *operando* Raman spectroscopy assisted by density functional theory (DFT) calculations using electrolyte salt concentrations between 0.3 and 7.0 molal (m) of the common LiTFSI salt dissolved in DME:DOL (1:1, v/v) and our previously proven *operando* Raman spectroscopy setup.^32^ In particular, PSs are selectively, by using confocal optics, detected in the separator facing the Li anode side, and thus, only diffused PSs are monitored.

## Experimental Section

### Electrolyte Preparation

LiTFSI (99.5%, Solvionic) was dried at 120 °C under vacuum for 24 h before use. 1,2-Dimethoxyethane, also known as monoglyme (DME, anhydrous, 99.5%, Sigma-Aldrich), and 1,3-dioxolane (DOL, 99.8% with 75 ppm of butylated hydroxytoluene as an inhibitor, Sigma-Aldrich) were both dried and stored in preheated (200 °C) 3 Å molecular sieves (Thermo Scientific Chemicals). LiTFSI was dissolved with an appropriate amount into a fixed volume of an equal volume of DME and DOL (1:1, v/v), followed by continuous magnetic stirring for 24 h at 25 °C to prepare 0.3–7.0 m electrolytes (Table S1). All preparation was made in an argon-filled glovebox (O_2_ and H_2_O levels < 1 ppm). The water content in the prepared electrolytes was between 28 and 40 ppm as determined by Karl Fischer titration.

### Carbon/Sulfur Composite Cathode Preparation

The C/S composite cathode with 60 wt % S loading was prepared as described previously.^12^ Briefly, sulfur (Sigma-Aldrich, 99.998% trace metal basis) and carbon black (Vulcan) were first mixed in a mortar before sodium carboxymethyl cellulose (Na-CMC, *M*_w_ = 700,000, Sigma-Aldrich) was added as a binder, with a weight ratio of 60:38.5:1.5, respectively. The mixture was then magnetically stirred to obtain a homogeneous slurry, which subsequently was cast on a 20 μm Al foil (Hohsen) to form a 250 μm thick coating using the doctor blade technique. After being coated, the electrode was dried at 60 °C under vacuum for 24 h.

### PS Solution Preparation

Solutions with nominal concentrations of 0.5 m PS (Li_2_S_4_, Li_2_S_6_, and Li_2_S_8_) were prepared by mixing Li_2_S (Sigma-Aldrich, 99.8%) and S powder in DME:DOL (1:1, v/v) to achieve the desired Li:S stoichiometries. The mixtures were stirred and heated at 60 °C for 72 h to ensure complete reactions. This resulted in clear Li_2_S_6_ and Li_2_S_8_ solutions but a suspension of Li_2_S_4_.

### *Operando* Raman Spectroscopy Experiments

#### *Operando* Cell Assembly

The Li–S battery cell was assembled using a spectroelectrochemical cell ECC-Opto-Std (EL-cell GmbH) in a sandwich configuration similar to a coin cell assembly. The cell included a ⌀10 mm C/S composite electrode containing ∼1 mg of S (1.3 mg_S_/cm^2^), one layer of a ⌀10 mm glass fiber separator (Whatman 1821 GF/B, 675 μm) containing 60 μL of electrolyte, and ⌀15 mm counter and reference electrodes of lithium metal (Toyota Tsusho, 200 μm) with a ⌀2 mm hole at the center. Here the electrolyte-to-sulfur ratio was 60 μL/mg_S_, much higher than that in practical cells but necessary in order to ensure reliable Raman signal acquisition and to minimize solvent evaporation. We do not believe the results to be totally and simply transferable or generic, but they do provide guidance. The cell used a borosilicate glass window to enable the observation of the Raman spectra. The cell assembly was performed in an Ar-filled glovebox with O_2_ and H_2_O levels < 1 ppm.

#### *Operando* Raman Measurements

All Raman spectra at 25 °C were collected on a LabRam HR Evolution (Horiba GmbH) spectrometer under confocal mode using a 633 nm He–Ne laser (∼3 mW) with an Edge filter, a 200 μm confocal hole, and a Syncerity OE detector. The laser was focused by a 10× and 50× lens for survey and high-resolution spectra, respectively, on the separator surface near the edge of the hole in the lithium metal anode to collect the spectral response of diffused PS species.

We first applied the same moderate spectral resolution (Δν ∼ 2.6 cm^–1^) that was previously proven to be sufficient to track PS evolution.^31–33^ All these survey spectra, covering 200–2400 cm^–1^, were collected in a single run using a 300 grooves/mm grating. Each spectrum is the average of 30 accumulations of 20 s of exposure. To resolve the details in the local coordination of TFSI, however, and correlate these to PS speciation and evolution, higher-resolution Raman spectra (Δν ∼ 0.3 cm^–1^) were needed and acquired. These experiments employed a higher groove density grating,[Bibr ref37] 1800 grooves/mm, to acquire 300–600 cm^–1^ for the PS species and 700–1000 cm^–1^ for the TFSI, DME, and DOL signals (and overall electrolyte). Each spectrum was acquired by adding 5 accumulations of 30 s of exposure, and the interval between measurements was 610 s, similar to the survey spectrum. Due to the low signal-to-noise ratio within the PS region with this setup, those species are primarily analyzed using the survey spectra.

#### Electrochemical Measurements

The assembled *operando* Li–S battery cell was directly transferred to the Raman instrument within ∼5 min to monitor the stability of the open circuit voltage (OCV) over a 20 min period. During this time, two Raman spectra were collected, each requiring 10 min to acquire at a resolution of 2.6 cm^–1^, in order to probe the possible changes in the electrolyte associated with the diffusion of PSs formed due to self-charge. After that, a galvanostatic measurement was performed using a constant current of 167 mA g^–1^, corresponding to a theoretical C/10 rate (1 C = 1672 mAh g^–1^), on a GAMRY Series G 300 instrument at 25 °C between 1.0 and 3.0 V vs Li^+^/Li^0^, and Raman spectra were continuously collected as described above.

#### Reference Raman Spectra

Both moderate- and high-resolution Raman spectra of the electrolytes and PS solutions were recorded on the same Raman spectrometer under the same confocal mode, as described above. An aliquot of the electrolytes or PS solutions was filled inside a 1.0 mm thick quartz cuvette (Hellma) under the argon atmosphere of the glovebox (O_2_ and H_2_O levels < 1 ppm). Then, the cuvette was sealed before it was transferred to the Raman spectrometer. The Raman spectra were acquired by focusing the laser into the solution with a 50× lens.

#### Spectra Analyses

First, the fluorescence background was corrected by using a polynomial function on the PS region (400–600 cm^–1^) and the electrolyte region (700–1100 cm^–1^) with Raman intensities initially offset to 0 at 600 and 1100 cm^–1^, respectively. Then, the spectral data were normalized by the standard normal variate method to have zero mean and normal standard deviation to account for random variances in the intensities. Subsequently, the PS region and the TFSI band (∼740–750 cm^–1^) were fitted with a linear combination of Gaussian–Lorentzian (Voigt) components. The initial time (*t* = 0) was set to when the second spectrum during OCV was acquired (ca. 20 min after start). These analyses were performed with MATLAB (version R2023b, The Mathworks, Inc.).

### Density Functional Theory Calculations

Density functional theory (DFT) calculations were performed to predict the stability of S_6_^2–^ toward homolytic dissociation into the S_3_^•–^ radical with and without being coordinated by Li^+^ and TFSI. Geometries of Li_2_S_6_, LiS_6_^–^, S_6_^2–^, S_3_^•–^, and LiS_3_^•^ species as well as Li–TFSI–PS clusters were optimized with the B3LYP functional^38,39^ and 6-311+g­(3df) basis set.^40^ To simulate solvation, we applied the implicit solvation model SMD^41^ based on DME parameters (Supporting Information). Local energy minima on the potential energy surface were obtained, as verified by having no imaginary vibrational frequencies. The dissociation of the central S–S bond was evaluated by the Gibbs free energy differences (Δ*G°*). All calculations were performed using the Gaussian 16 program package.^42^

## Results and Discussion

Indeed, a higher electrolyte lithium salt concentration can effectively decrease both sulfur and PS solubility (Table S2).^12^ Here, to qualify and semiquantify the practical benefits, we monitor PS species using *operando* spectroscopy of Li–S cells, first under OCV conditions, and subsequently we resolve how the salt concentration controls PS evolution, solubility, diffusion, and reactions upon cycling.

### OCV Measurements

The OCV of the cell with the 0.3 m electrolyte, directly after assembly, is constant at ∼2.4 V, while the 1.0 m electrolyte cell shows the loss of voltage, from ∼2.9 V to a stable value of 2.4 V within 20 min (Figures 1a and S1). The cell with the 2.0 m electrolyte shows a more gradual loss of voltage. Further increasing the electrolyte concentration to 3.0, 5.0, and 7.0 m helps to maintain the OCV at around 3.0–3.2 V, of which those of the two latter electrolytes are slightly lower (∼1.5%) than that of the 3.0 m electrolyte. We attribute the overall increase in the value and stability of OCV in the 0.3–7.0 m electrolytes to both (i) changes in Li^+^ solvation structure and ion speciation to more CIPs and AGGs^12,43^ and (ii) the reduction of the solubility and diffusion of sulfur at higher concentrations.^12^ The slight invert trend in the 3.0–7.0 m electrolytes, on the other hand, could be due to the lower ionic conductivity and higher viscosity of highly concentrated electrolytes,^12^ of which the latter could affect electrode wetting.

**Figure 1:**
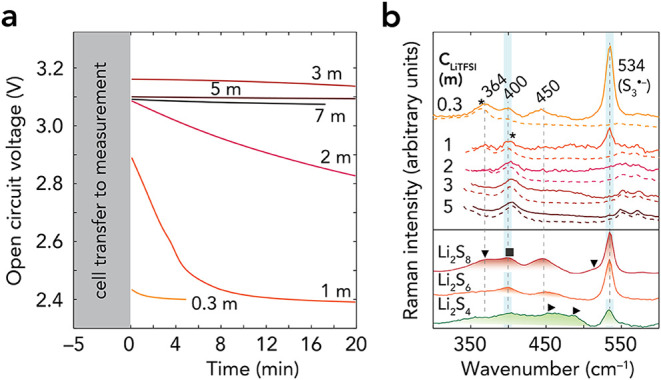
(a) OCV of Li–S *operando* cells using 0.3–7.0 m electrolytes. Repeating OCV measurements are shown in Figure S1. (b) Comparison of electrolyte Raman spectra (solid color lines) with pristine electrolytes (dashed color lines) and Li_2_S*_n_* (*n* = 4, 6, 8) in DME:DOL solutions (color lines with shading). Symbols: (*) DME vibrations, (▼) S_8_^2–^ at 369 and 511 cm^–1^, (▶) S_4_^2–^ at 450 and 468 cm^–1^, and (■) S_*n*_^2–^ at 400 and 450 cm^–1^. A detailed assignment can be found in Table S3.

The lower solubility and diffusion of sulfur should decrease the concentration of PSs chemically formed at the anode. To detect these PS species, we probe the S–S stretching vibrations (300–600 cm^–1^) (Figure 1b). New bands arise at 400, 450, and 534 cm^–1^ in the 0.3 m electrolyte cell, alongside the vibrational signatures of TFSI and solvents (Table S3). The 400 and 450 cm^–1^ bands correspond to S–S vibrations from various long and short PS S_*n*_^2–^ (*n* = 4–8).^32,44,45^ The 534 cm^–1^ band is due to the S_3_^•–^ radical,^32,44,46^ whose first overtone appears at 1068 cm^–1^ (Figure S2). S_3_^•–^ is the product of the homolytic dissociation of S_6_^2–^ (S_6_^2–^ ⇌ 2S_3_^•–^), as confirmed by methods such as electron paramagnetic resonance^47^ and UV–vis spectroscopy.^48^ The main band at 369 cm^–1^ of S_8_^2–^ is, on the other hand, difficult to distinguish from the bands of DME and TFSI. Still, the stable voltage at 2.4 V and the absence of the S_4_^2–^ vibration at 468 cm^–1^ support the idea that long-chain PSs, such as S_6–8_^2–^, are predominant. To the best of our knowledge, these peaks are mainly assigned to free, fully solvent-separated dianion (S_*n*_^2–^)/radical (S_3_^•–^) species,^45^ rather than CIP- or AGG-type PSs.

Using the 1.0 m electrolyte, we could not clearly distinguish the bands corresponding to PS dianions, such as 400 and 450 cm^–1^, from those of the TFSI anion (Figure 1b and Table S3), while the 534 cm^–1^ band appears with weak intensity. This suggests that the PS concentration is below the detection limit, which agrees with slower voltage decay. The signal of S_3_^•–^ is, on the other hand, enhanced (by 10^3^–10^5^ times) through resonance Raman conditions occurring when matching its electronic transition (617–627 nm)^35,49^ with our 633 nm excitation laser wavelength, even though low-donor solvents like DME and DOL do not stabilize the radical.^50^ Thus, we can still detect S_3_^•–^ despite its low concentration. When the cell was left resting at OCV for a more extended 5 h period, the PS dianion (369, 400, and 450 cm^–1^) and S_3_^•–^ (534 cm^–1^) bands continuously grew in intensity (Figure S3). When moving to the 2.0–5.0 m electrolytes, we could not detect any PS signals, suggesting slower chemical reduction kinetics of sulfur. These slower kinetics, in turn, align with less dissolved sulfur due to the lower solubility limit (Table S2) and higher viscosity of these electrolytes.^12^ As a consequence, it assists to maintain the OCV > 2.8 V (Figure 1a), with some notable changes for the 2.0 m electrolyte, by decreasing the spontaneous reactions between dissolved sulfur and the lithium anode. The self-discharge thus slows as the electrolyte salt concentration increases.

### *Operando* PS Evolution

During discharge, the Raman spectra of the cells using the 0.3 m (Figures 2a and S4) and 1.0 m (Figures 2b and S5) electrolytes reveal systematic changes in the PS dianion and radical bands during the 2.4 V plateau, in agreement with a previous study by some of us.^32^ The 400 and 450 cm^–1^ bands, which contain mixed contributions from S_8_^2–^, S_6_^2–^, and S_4_^2–^ (c.f. Figure 1b) depending on the cell SOC, are dominated by S_6_^2–^ at the end of the 2.4 V plateau,^51^ as confirmed by the concurrent growth of the S_3_^•–^ band at 532 cm^–1^. As the discharge proceeds into the 2.1 V plateau (*t* = ∼2–6 h), these same bands decrease in intensity, reflecting the consumption of S_6_^2–^ and the emergence of shorter PSs (e.g., S_4–5_^2–^) as the dominant contributor. In parallel, the S_3_^•–^ signal weakens but remains detectable, which can be explained by the dissociation of unconverted S_4_^2–^ (S_4_^2–^ ⇌ 2S_2_^•–^) and subsequent comproportionation (S_4_^2–^ + S_2_^•–^ ⇌ S_3_^•–^ + S_3_^2–^) as proposed by Steudel et al.^52^ However, no clear spectroscopic evidence for S_2_^•–^ (e.g., a resonance-enhanced band at 582 cm^–1^)^53^ was obtained under our Raman conditions, and the bands of unstable S_3_^2–^ (e.g., 238 and 466 cm^–1^)^54,55^ might be at low intensity and hence are potentially overlapped with those of S_4–5_^2–^. We also note here that for the cell with the 0.3 m electrolyte, solid α-S_8_ appears after the eighth hour of cycling with strong peaks that overlap with the PS signals (Figure S4), possibly due to PS decomposing after long laser exposure.

**Figure 2:**
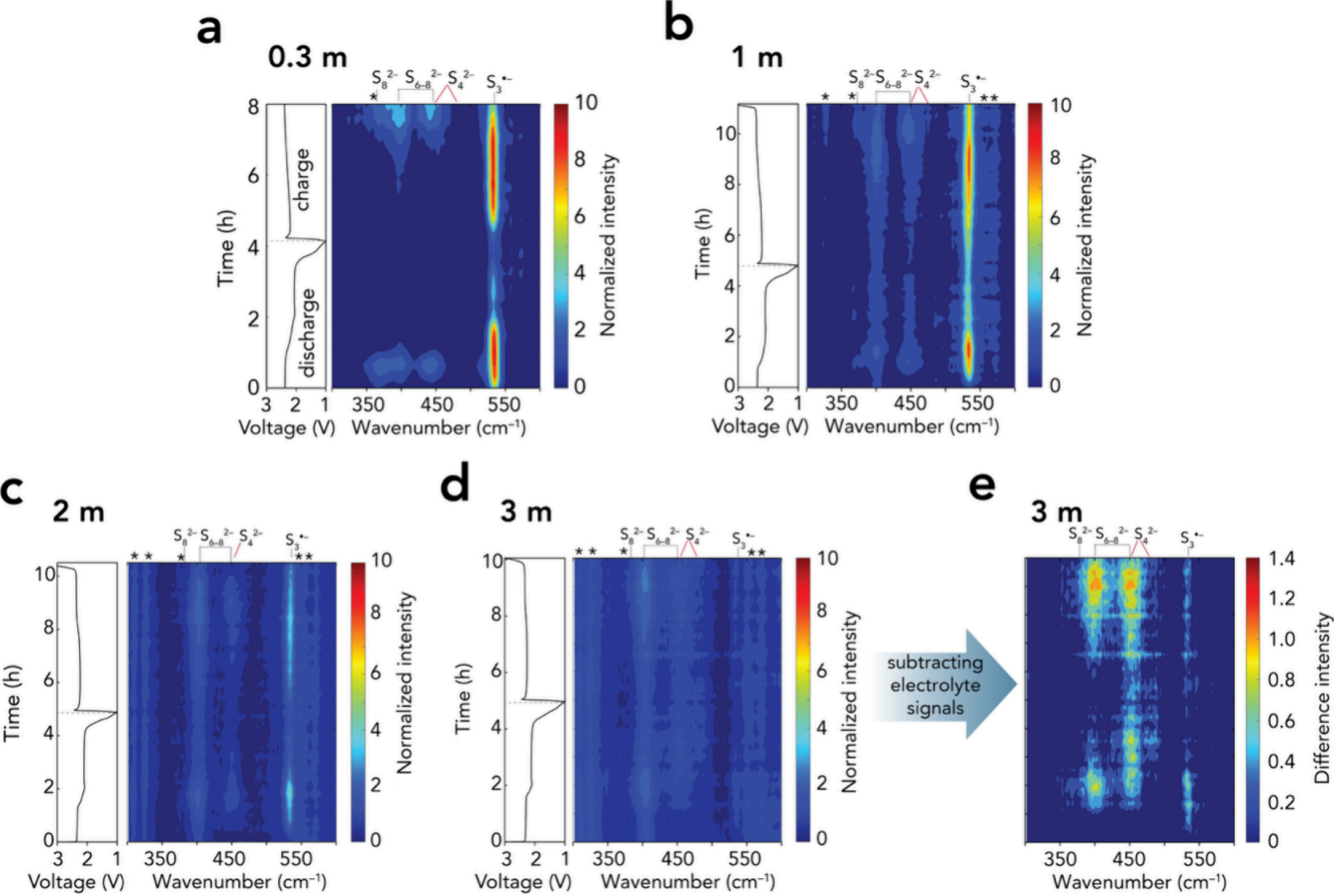
*Operando* Raman spectra as heat maps coupled with cell voltage profiles for cells with (a) 0.3, (b) 1.0, (c) 2.0, and (d) 3.0 m electrolytes. (e) Difference spectra of the data in (d) remove the contribution of electrolyte-related vibrations to better recover PS signals. (Full spectral ranges are shown in Figures S4 and S5.) Symbols: (*) vibrations from TFSI and the solvents (c.f. Table S3). Note that the peak of TFSI at 400 cm^–1^ remains as the background in (b–d).

Using the 2.0 m electrolyte (Figures 2c and S5), the PS bands at 400, 450, and 534 cm^–1^ are weaker. They are even almost identical to the TFSI bands when using the 3.0 m electrolyte (Figures 2d and S5), thus we can only resolve them by difference spectra (Figure 2e). Notably, S_8_^2–^ (369 cm^–1^) is unresolved in Figure 2c–e, perhaps due to its limited concentration, and this gives the most substantial evidence that bulky PSs diffuse much more slowly in highly viscous electrolytes.

Moving to the 5.0 and 7.0 m electrolytes, we do not detect the presence of either PS dianions or the S_3_^•–^ radical (Figure S6). Furthermore, the electrochemical behavior differs, typically lacking the 2.4 V plateau, and the latter cell completes the initial discharge quickly in less than 2 h. We attribute their poor electrochemical performance to sluggish sulfur utilization kinetics, given also that Figure S7 shows hampered sulfur conversion, which contrasts with some earlier reports^10,20^ but aligns with the observations by Shen et al.,^19^ likely due to differences in cathode design. Nonetheless, the stable OCV (c.f. Figure 1a) and the absence of detectable PSs suggest that both sulfur and PSs remain undissolved and do not migrate to the anode.

### Raman Intensity and Voltage Profiles

To further correlate the effect of salt concentration on selectively controlling PS dissolution, diffusion, and reactivity, we analyze both the Raman intensity profiles of PSs and the voltage profiles in more detail. First, the distinct two-plateau discharge profiles (Figure 3a) show improved sulfur utilization as more capacity is delivered as a function of salt concentration, up to 3.0 m. The charge profiles (Figure 3a), in turn, suggest a reduced overcharge for the 3.0 m electrolyte cell as compared with the other cells.

**Figure 3:**
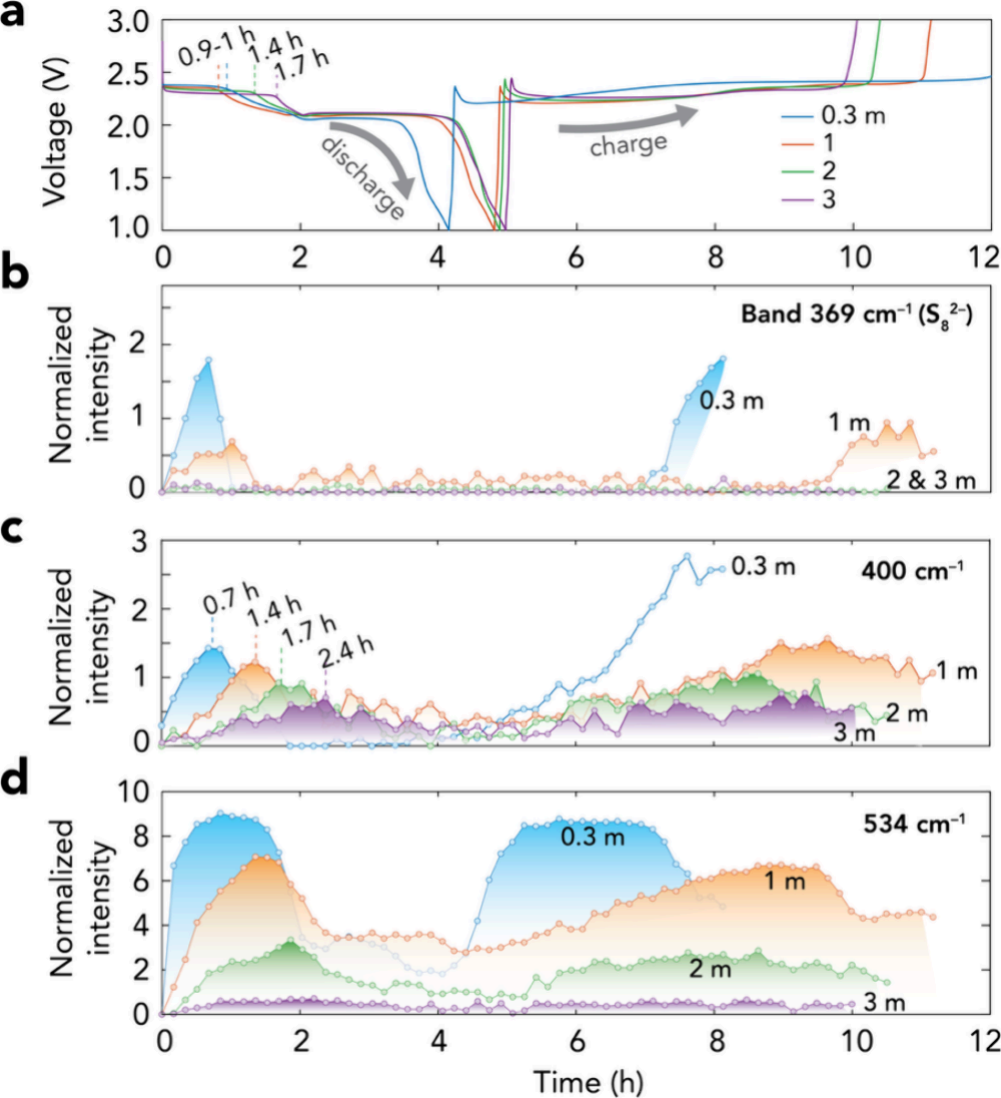
(a) Voltage profiles and (b–d) Raman intensity profiles of PSs at (b) 369, (c) 400, and (d) 534 cm^–1^ in Li–S battery cells using the 0.3–3.0 m LiTFSI electrolytes.

#### Dissolution and Diffusion of PSs

The length of the 2.4 V discharge plateau suggests that more long-chain PSs form as a function of increasing salt concentration. However, the intensity profiles show quite the opposite: a lower concentration and slower growth rate (Figure 3b,c). Here, notable changes in the Raman intensity reflect only soluble PSs that diffuse, while insoluble/nondiffused species remain undetected. This leads to (i) the absence of bulky S_8_^2–^ (369 and 511 cm^–1^, Figure 3b) and (ii) a decrease in shorter PS species (*n* = 4–6) at 400 cm^–1^ (Figure 3c) and 450 cm^–1^ (Figure S8).

The slower formation/migration of PSs is further revealed by the delayed maxima of the 400 cm^–1^ (Figure 3c) and 450 cm^–1^ (Figure S8) bands, i.e., not always coinciding with the end point of the 2.4 V plateau, proposed to be the complete reduction of S/longer PS to S_6_^2–^.^4^ The 2.4 V end point shifts from ∼0.9 to 1 h (0.3 and 1.0 m) to 1.4 and 1.7 h (2.0 and 3.0 m, respectively) (Figure 3a), while the 400 cm^–1^ maximum (Figure 3c) occurs at ∼18 min before this point using the least concentrated electrolyte but shifts to 18 and 42 min later. We attribute these delays to slower PS diffusion in the more concentrated electrolytes, though more complete S/PS conversion, and thus slower chain-length speciation, may also contribute due to the higher capacities extracted.

The fast decrease observed for both the 400 and 450 cm^–1^ bands right after the beginning of the 2.1 V plateau does not, however, indicate fast reduction of S_6_^2–^ or S_4_^2–^. This is rather influenced by (i) the inherently weak Raman activity of S_4_^2–^ as compared to long-chain PSs^44^ and (ii) the limited presence of S_4_^2–^ due to its extremely low solubility.^5^

On charge, the PS profiles show a decline in the total PS concentration as a function of electrolyte salt concentration. Shorter 2.4 V charge plateaus suggest that the PS maxima should follow the opposite trend of the discharge. This is true for the 1.0 and 2.0 m electrolyte cells, while for the 3.0 m electrolyte cell, the maximum could not be unambiguously identified. For the cell with the 0.3 m electrolyte, the PS intensity profiles are incomplete due to early deposition of solid α-S_8_, whose Raman signals overlap with other PS bands (Figure S4) as mentioned above.

#### S_3_^•–^ Radical Formation

While we expect the 534 cm^–1^ band of the S_3_^•–^ radical to grow alongside other bands of PSs, especially the 400 cm^–1^ band, this is not always the case across all electrolyte salt concentrations (Figure 3d). It shows a quite stable intensity for ∼1 and ∼2.5 h after reaching its maximum during discharge and charge. The growth seems faster than the growth of other PS bands, especially that of S_6_^2–^ (400 cm^–1^), for the cell with the least concentrated electrolyte during both processes. For the 1.0 and 2.0 m electrolyte cells, the 534 cm^–1^ band growth is more synchronous with that of the S_6_^2–^ band and exhibits clear maxima, while it becomes too small to assess in the cell with the 3.0 m electrolyte.

To illustrate further how the salt concentration affects the S_3_^•–^ radical increase, we compare the intensity profiles of the 400 and 534 cm^–1^ bands (Figure 4). The two corresponding species increase simultaneously but at different rates. This is evident through the considerable intensity loss of S_3_^•–^ compared to that of S_6_^2–^(Figures 4a,b), and the ratio (Figure 4c) reveals that S_6_^2–^ is predominant over S_3_^•–^, especially considering the predicted Raman activity of S_3_^•–^ to be ca. 2 to 3 orders of magnitude larger than that for S_6_^2–^, strongly suggesting that the S_3_^•–^ concentration likely is negligible at high electrolyte salt concentrations.

**Figure 4:**
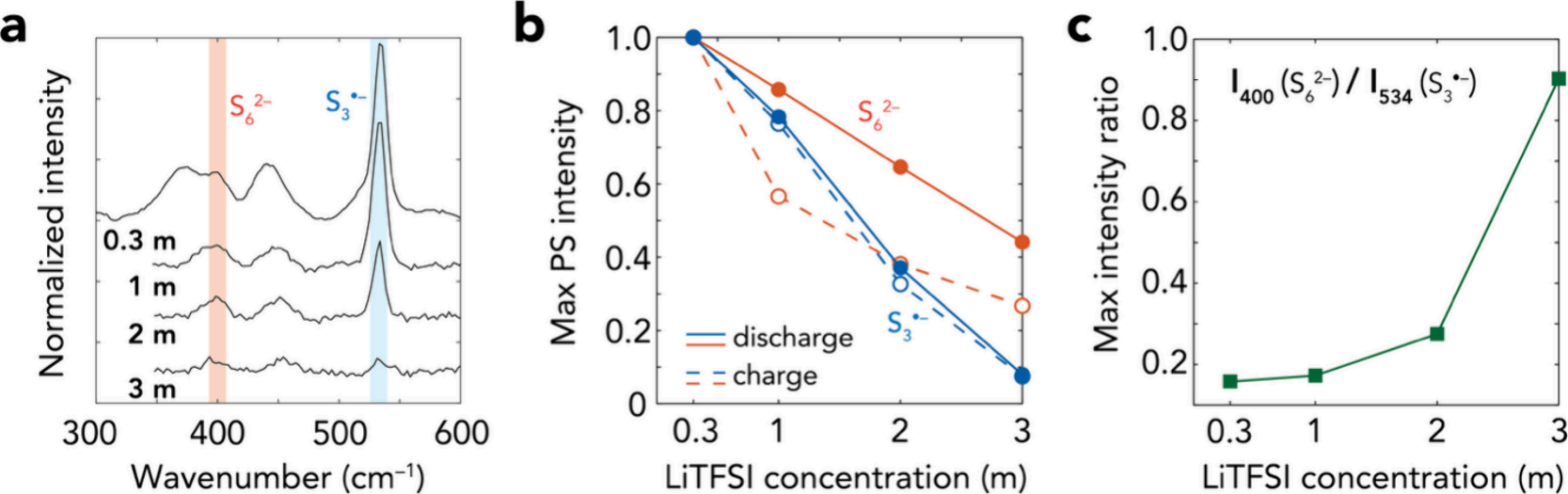
(a) Selected Raman spectra for when the 400 cm^–1^ band achieves maximum intensity. (b) The normalized maxima of S_6_^2–^ (400 cm^–1^) and S_3_^•–^ (534 cm^–1^) intensities in these cells during discharge and charge. (c) The ratio between the maxima of S_6_^2–^ and S_3_^•–^ intensities during cell discharge.

The comparison between the 400 and 534 cm^–1^ bands also suggests that the S_3_^•–^ radical formation mechanism has been altered. Although the canonical S_6_^2–^ → 2S_3_^•–^ pathway is strongly exergonic (Table 1; also see ref 52) to generate abundant S_3_^•–^, experiments have shown nondetectable to trace amounts in many solvents.^35,50,56^ Previous modeling has suggested solvent-dependent dissociation pathways to S_6_^2–^, and subsequently the S_3_^•–^ radical, that are unfavorable in DME and DOL.^28,56^ High salt concentrations could additionally render strong PS–Li–TFSI interactions^30^ (Figure S9), and we hypothesize that these can potentially be formed in our 3.0 m electrolyte, where the DME:Li ratio is low (Table S1), whereas DOL is less active^43^ and hence not capable to fully solvate Li^+^. These interactions could provide an extra hindrance for the cleavage of the central S–S bond of S_6_^2–^, as evidenced by the barely favorable Δ*G°* for LiS_6_^–^ and unfavorable Δ*G°* for Li_2_S_6_ and Li_3_[(TFSI)­S_6_] (Table 1). Still, it remains elusive how suppressing the S_3_^•–^ radical significantly alters the PS redox mechanism(s), especially since several studies suggest that this radical accelerates S/PS conversion.^46,50,57^

**Table 1:** DFT Calculated Δ*G°* (kJ/mol) from Structures (Figure S9) Optimized at the B3LYP/6-311+g­(3df) Level of Theory and Implicit Solvation (SMD) Using DME Parameters

No.	Reaction	Δ*G°*
1	S_6_^2–^ → 2S_3_^•–^	–71.6
2	LiS_6_^–^ → Li[(S_3_^•^)_2_]^−^	–4.4
3	Li_2_S_6_ → Li_2_[(S_3_^•^)_2_]	25.7
4	Li_3_[(TFSI)S_6_] → Li_3_[(TFSI)(S_3_^•^)_2_]	37.4

### *Operando* TFSI Local Environment Changes

Probing the PS–Li–TFSI interactions in the 3.0 m electrolyte using *operando* high-resolution Raman spectroscopy shows that the overall spectra appear almost unchanged on a cursory inspection (Figure 5a). However, deconvoluting the TFSI band (740–750 cm^–1^), which is far more responsive to solvation variations^22^ than the PS bands (300–600 cm^–1^), provides some further insight (Figure S10). The deconvolution renders a 741 cm^–1^ component for “free” TFSI (SSIPs) and a 747 cm^–1^ component for Li^+^–TFSI (CIPs), in which the latter is more populated than the former as salt concentration increases (Figure S10).^22^ Upon cycling, the SSIP/CIP intensity ratio inversely follows the changes in concentrations of PSs at different SOC, repeating for five cycles, and simultaneously declines steadily, largely from solvent evaporation (similarly to the pristine electrolyte) (Figures 5b,c and S11). We ascribe the minor shift from SSIPs to CIPs on the 2.4 V plateau to changes in the solvation of TFSI due to the addition of Li^+^ from the anode and long-chain PSs. We could also assign the decreased SSIP/CIP ratio to the formation of TFSI–Li–PS species, adapting the common observation that PS–Li^+^ interactions are inevitable.^26,30^ PS–Li^+^ interactions become stronger as short-chain PSs (S_*n*_^2–^, 1 < *n* < 5) are produced on the 2.1 V plateau, which will eventually overcome the Li^+^–TFSI interactions, consequently releasing free TFSI anions and thus raising the SSIP/CIP ratio. Collectively, these data provide indirect, yet compelling, evidence of a dynamic Li^+^, TFSI, and PS interplay (solute–solute interactions) throughout the entire operation of the Li–S battery cell (Figure 5d).

**Figure 5:**
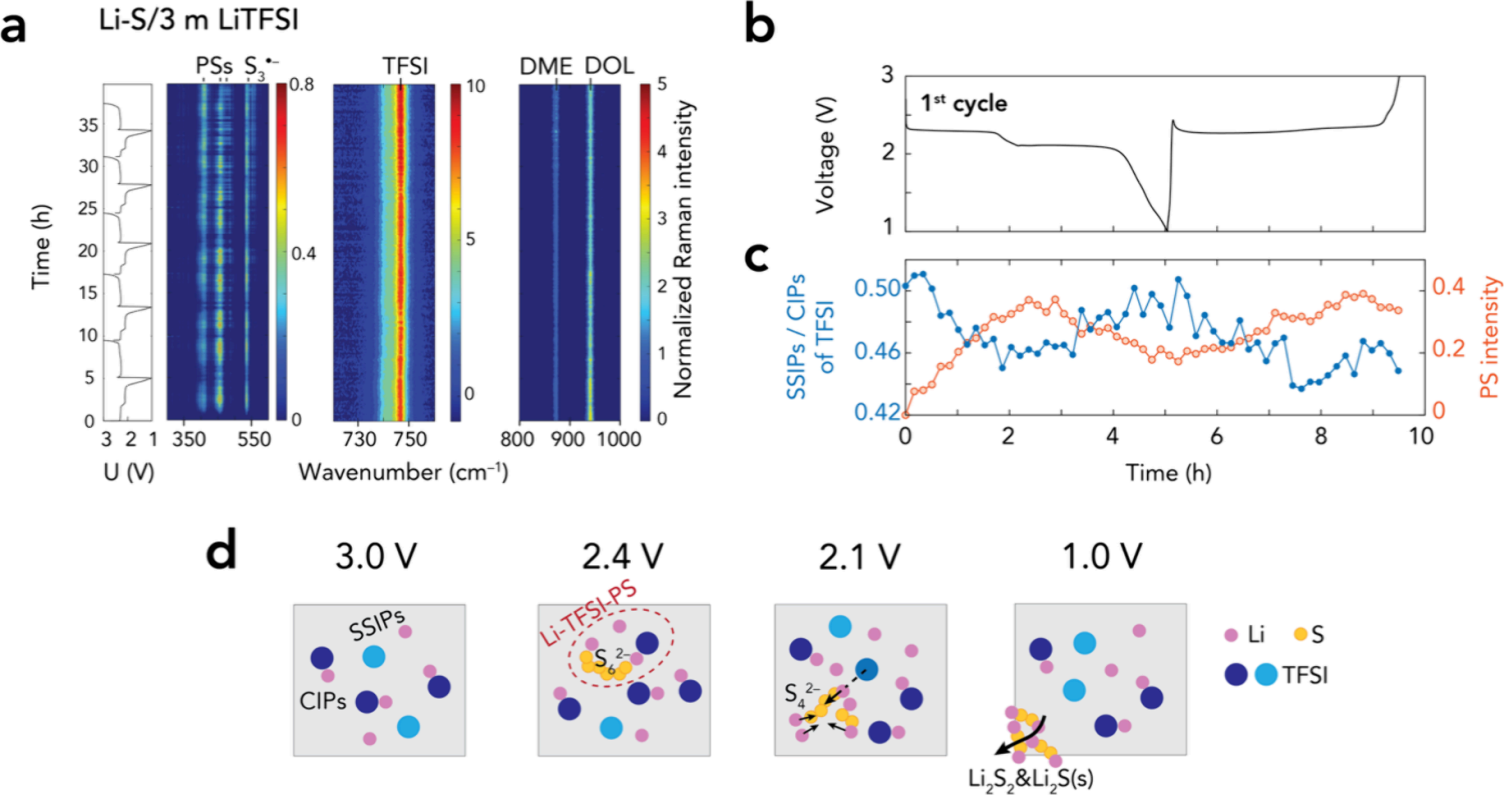
(a) *Operando* high-resolution Raman spectra as heat maps coupled with cell voltage profiles for the cell using the 3.0 m LiTFSI electrolyte. (b) The voltage profile of the first cycle and (c) Raman intensity profiles of SSIPs/CIPs and PS (the 400 cm^–1^ band). (d) Schematic representation of the dynamic speciation and interaction between Li^+^, TFSI, and PSs in the electrolyte of a Li–S battery cell at different SOC.

## Conclusions

By using *operando* Raman spectroscopy, we directly illustrate that both S and PS dianions (S_4–8_^2–^) are less soluble and diffuse slower to the lithium anode when increasing the electrolyte salt concentration from 0.3 to 3.0 m, thereby improving the battery performance. In the 5.0 and 7.0 m electrolytes, however, S and PSs are in principle insoluble and do not migrate to the anode due to their high viscosities, causing sluggish S/PS conversion; this also results in poor electrochemical performance. The Raman spectra also show that the S_3_^•–^ radical is suppressed in the more concentrated electrolytes, an effect attributed to Li–TFSI–PS interactions that lead to a less favorable/unfavorable S_6_^2–^ disproportionation pathway, as supported by the DFT calculation. These species and interactions can indirectly be monitored via high-resolution Raman signatures of the TFSI anion, which can reveal how salt concentration controls the solvation and thereby dictates both the chemistry and the macroscopic transport properties of PSs. Overall, the here showcased real-time tracking of the solubility, mobility, and interconversions of PSs provides knowledge highly essential for guiding further advances in Li–S batteries.

## Supplementary Material



## Notes

Details of solvent descriptors for DFT calculation, extra data of OCV measurements, raw and processed Raman spectra of all *operando* experiments, Raman spectra for the *operando* experiments at the sulfur cathode, and DFT optimized geometries (PDF)
