# Supplementary material for: Salt Concentration Control of Polysulfide Dissolution, Diffusion, and Reactions in Lithium–Sulfur Battery Electrolytes
Source: ACS Appl Energy Mater. 2025 Oct 31;8(21):15830–7. doi: 10.1021/acsaem.5c02378 (PMC12606560; doi:10.1021/acsaem.5c02378)
Supplement: Supplementary file 1 [file ae5c02378_si_001.pdf]

SUPPORTING INFORMATION

Salt concentration control of polysulfide dissolution, diffusion and reactions in lithium-sulfur battery electrolytes

N. Tan Luong,[1] Aginmariya Kottarathil,[1,2] Władysław Wieczorek[2,3] and Patrik Johansson[1,3]*

[1] Department of Physics, Chalmers University of Technology, 412 96 Gothenburg, Sweden.

[2] Faculty of Chemistry, Warsaw University of Technology, 00664 Warzaw, Poland.

[3] ALISTORE—European Research Institute, CNRS FR 3104, Hub de l'Energie, 15 Rue Baudelocque, 80039 Amiens, France

***Corresponding Author: tan.luong@chalmers.se**

Revision submitted to ACS Applied Energy Materials

Date: September 29th, 2025

Solvent descriptors for SMD model

To define the DME solvent using the SMD model in Gaussian 16, the following input file was used:

```
#p opt=calcfc freq=raman
b3lyp/6-311+g(3df)
scrf=(smd,solvent=generic,read)
geom=connectivity
gfoldprint
scf=maxcycle=1000

[Molecular details:
title, charge, multiplicity, coordinates]

stoichiometry=C4O2H10
solventname=DimethoxyEthane
eps=7.55
epsinf=1.896129
hbondacidity=0.00
hbondbasicity=0.68
SurfaceTensionAtInterface=35.4216652
CarbonAromaticity=0.0
ElectronegativeHalogenicity=0.0
```

The solvent descriptors (refractive index; Abraham's hydrogen bond acidity and basicity parameters; macroscopic surface tension; dielectric constant at 298.15 K) for DME were obtained from a previous study,[1] and cross-checked with other literature.[2, 3]

FIGURES

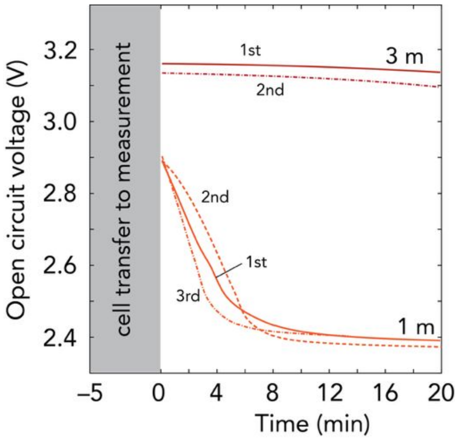

Figure S1. Reproducibility trend of OCVs is shown in the Li-S battery cells using 1 and 3 m LiTFSI in DME:DOL (1:1, v/v).

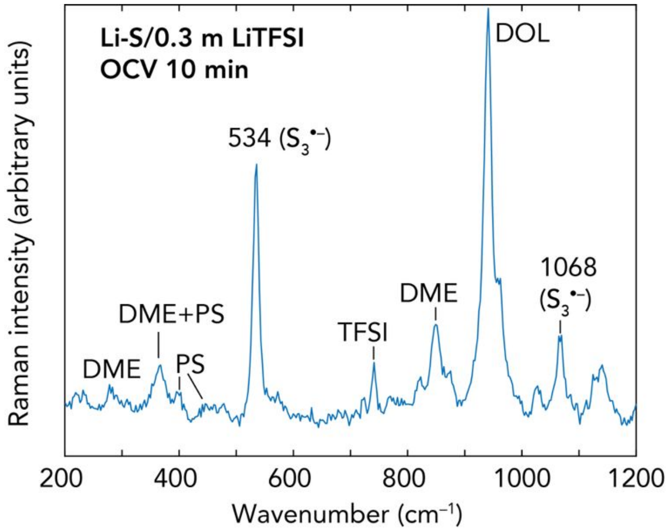

Figure S2. Raman spectrum of the Li-S battery cell using 0.3 m LiTFSI in DME:DOL (1:1, v/v) after 10 min of resting at OCV, showing the peaks of PSs at 369 (potentially overlapped with DME), 400, and 450 cm^{-1}, and the $S_3^{\bullet -}$ radical at 534 and 1068 cm^{-1}.

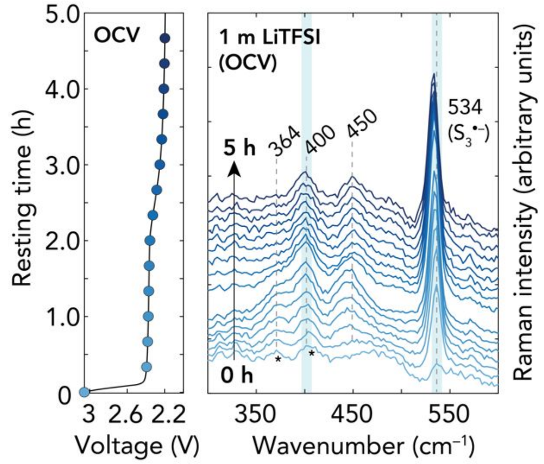

Figure S3. Sequence of Raman spectra of the Li-S battery cell using 1 m LiTFSI in DME:DOL (1:1, v/v) during 5 h of resting at OCV. Left panel: Voltage profile. Right panel: Development of Raman peaks associated to various PSs (Li_2S_{4-8}). The asterisks (*) mark the overlapping peaks of PSs and electrolyte.

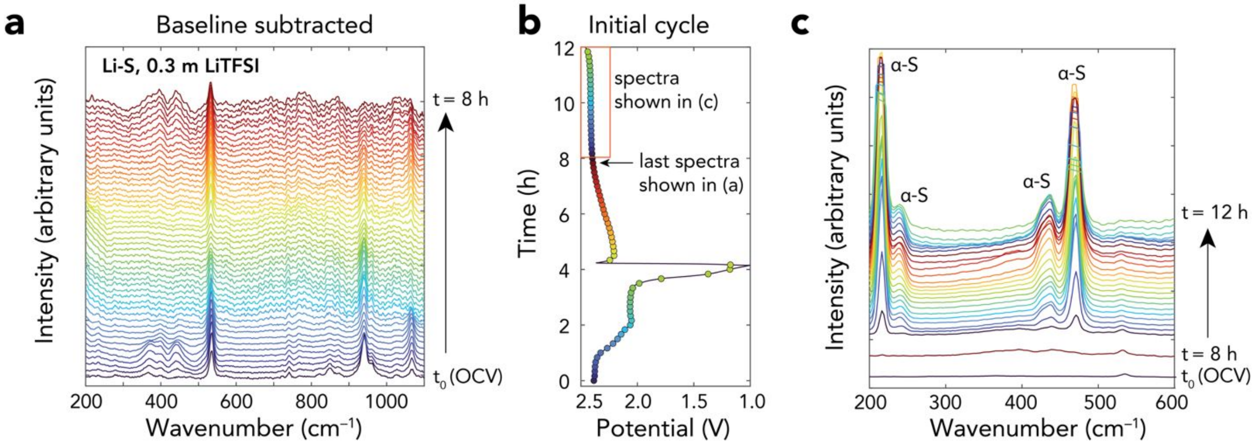

Figure S4. *Operando* Raman experiments of the Li-S battery cell using 0.3 m LiTFSI in DME:DOL (1:1, v/v). (a) Raman spectra collected from 0 (OCV) to 8 h, corresponding to voltage profiles in (b). (c) Spectra after 8 h until finished cycling with strong peaks of solid α-S_8 and weaker PS peaks.

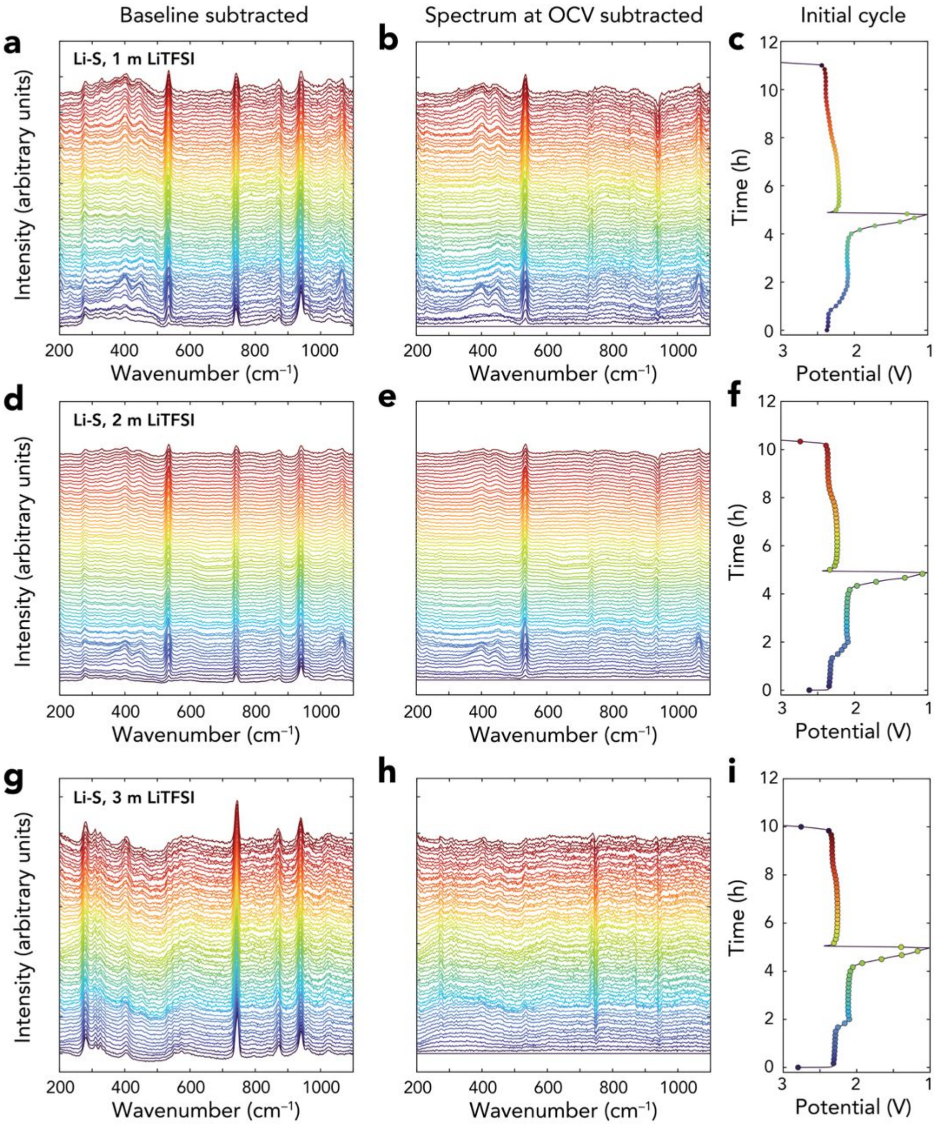

Figure S5. *Operando* Raman experiments of Li-S battery cells using (a–c) 1 m LiTFSI, (d–f) 2 m LiTFSI, and (g–i) 3 m LiTFSI in DME:DOL (1:1, v/v) electrolytes. (a, d, g) Baseline-subtracted Raman spectra, (b, e, h) difference spectra from the spectrum at OCV, and (c, f, i) the corresponding voltage profiles of the first discharge/charge cycle.

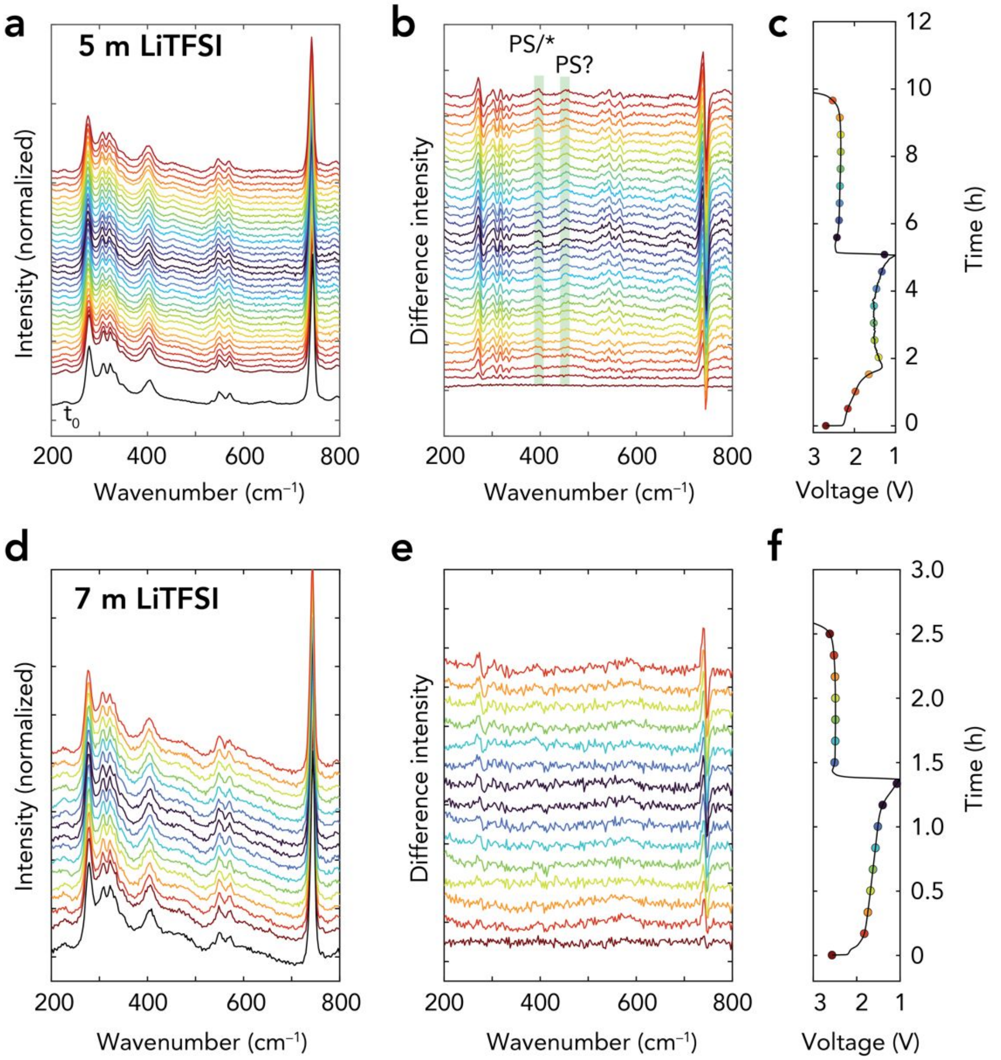

Figure S6. *Operando* Raman experiments of Li-S battery cells using (a–c) 5 m LiTFSI and (d–f) 7 m LiTFSI in DME:DOL (1:1, v/v) electrolytes. (a, d) Baseline-subtracted Raman spectra, (b, e) difference spectra from the spectrum at OCV, and (c, f) the corresponding voltage profiles of the first discharge/charge cycle.

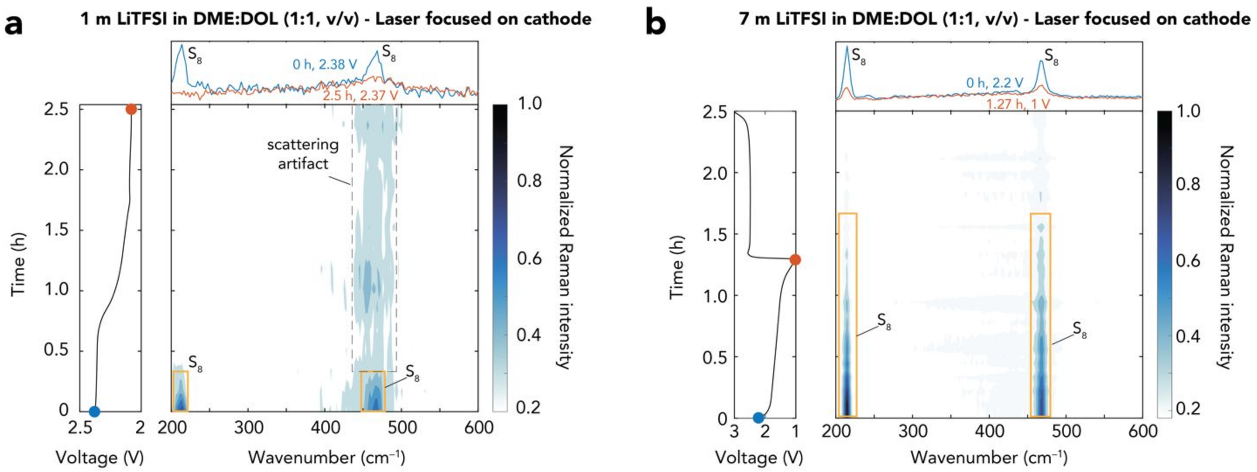

Figure S7. *Operando* Raman experiments Li-S battery cells using (a) 1 m LiTFSI and (b) 7 m LiTFSI in DME:DOL (1:1, v/v) electrolytes with the laser focused on the C/S cathode, showing the loss of α-S_8 upon discharge for 2.5 h. While S_8 totally disappears in (a) after ~30 min ($U = 2.4$ V), it still remains with a discernible concentration in (b). The cell using the 7 m electrolyte completes the discharge after only 1.3 h, showing poor capacity and sluggish kinetics.

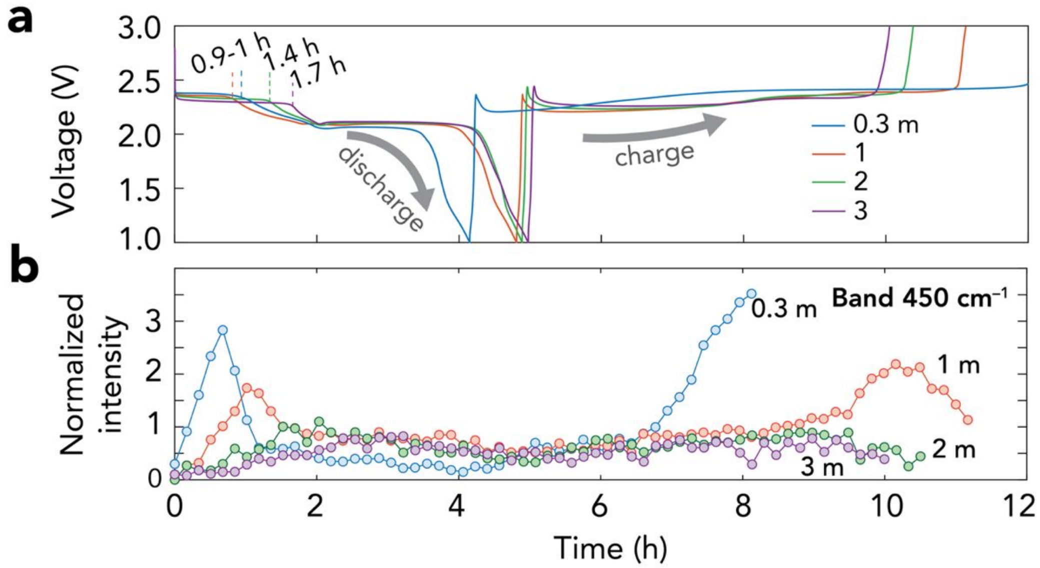

Figure S8. (a) Voltage and (b) intensity profiles of PSs at 450 cm^{-1} of Li–S battery cells cycled at C/10 using 0.3–3 m LiTFSI in DME:DOL (1:1,v/v) electrolytes.

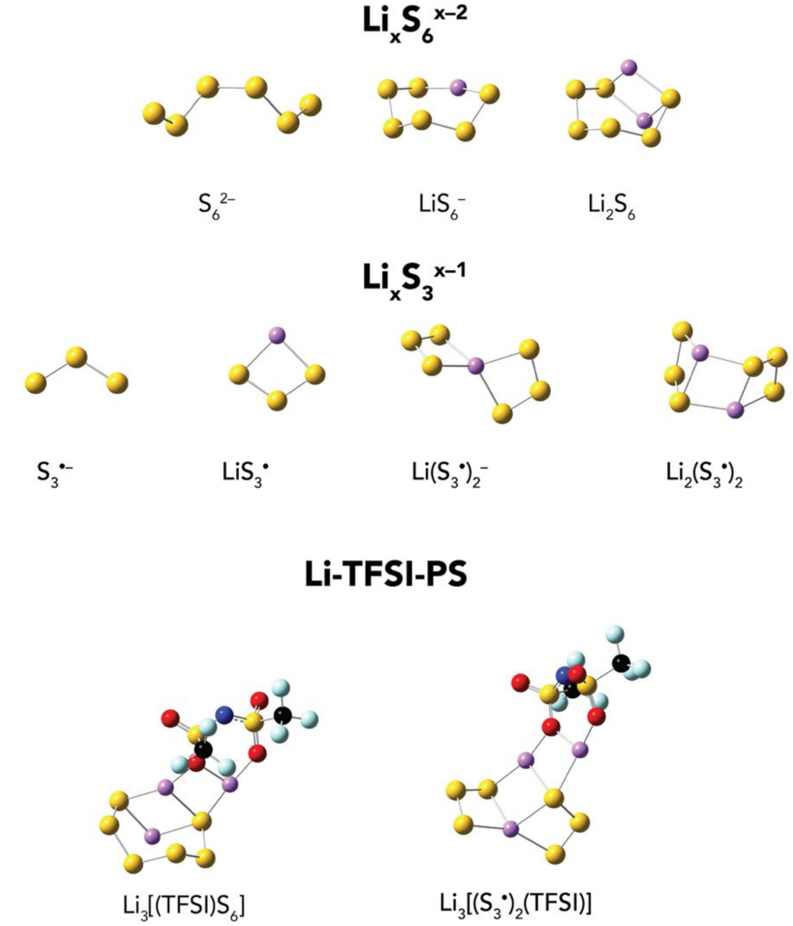

Figure S9. DFT optimized geometries of different TFSI–Li–PS species obtained using B3LYP/6–311+g(3df) level of theory and implicit solvation by the SMD model and parameters for DME.

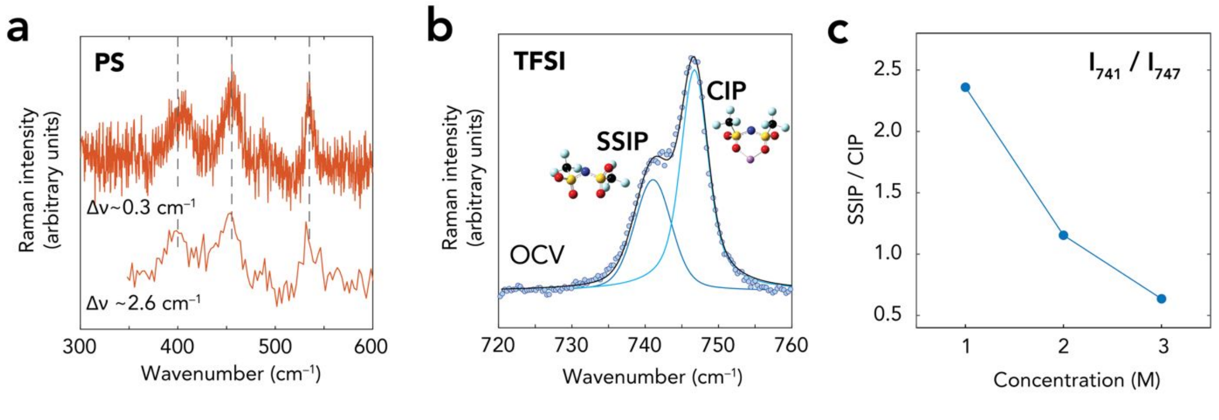

Figure S10. (a) Comparison between selected high- and low-resolution Raman spectra (t = 2.88 h) from the *operando* experiments using the 3 m electrolyte. (b) A selected high-resolution Raman spectrum of the TFSI band (at OCV) of the 3 m electrolyte and its deconvolution into SSIP and CIP peaks. (c) Relative SSIPs/CIPs ratio derived from the deconvolution of Raman spectra of the pristine 1–3 m electrolytes.

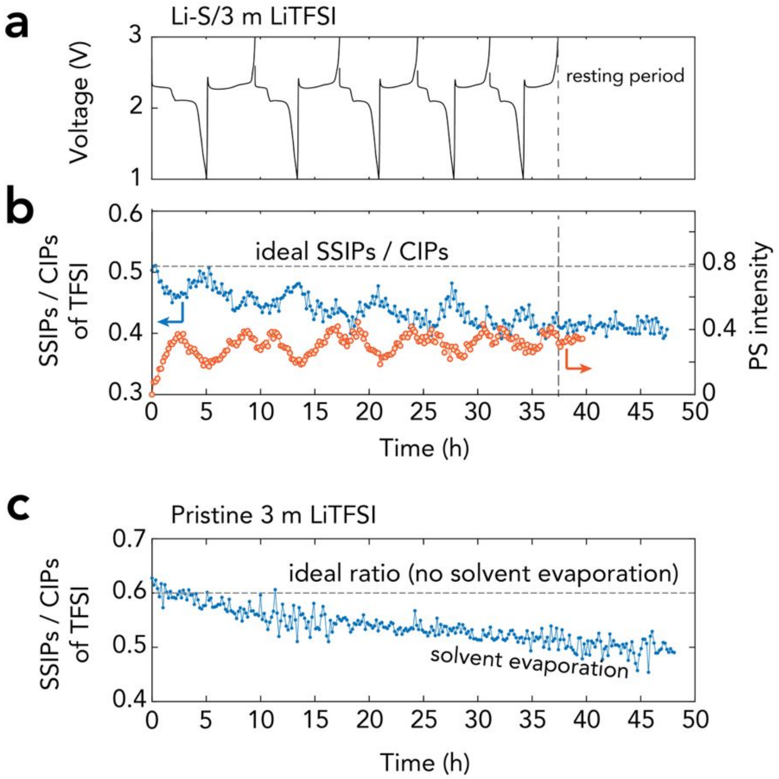

Figure S11. (a) Voltage profiles and (b) Raman intensity profiles of SSIPs/CIPs and PSs over 5 cycles. (c) Raman intensity ratio of SSIPs/CIPs in pristine electrolyte, repeatedly collected in the *operando* Raman cell (ECC-Opto-Std, EL-cell® GmbH) for 48 h.

TABLES

Table S1. Electrolyte compositions.

Concentration (m)	n_{Li} (mol)	n_{DME} (mol)	n_{DOL} (mol)	Molar ratio DME:Li	Molar ratio DOL:Li
0.3	0.0003	0.0044	0.0072	14.8	23.8
1	0.001	0.0044	0.0072	4.4	7.2
2	0.002	0.0044	0.0072	2.2	3.6
3	0.003	0.0044	0.0072	1.5	2.4
5	0.005	0.0044	0.0072	0.9	1.4
7	0.007	0.0044	0.0072	0.6	1.0

Table S2. Solubility data of sulfur S_8 (experimental) and Li_2S_8 (predicted) as a function of LiTFSI concentration (C_{LiTFSI}) in DME:DOL (1:1 v/v). These data were adapted from previous work.[4]

C_{LiTFSI} (m)	S_8 (mg S·L^{-1})	Li_2S_8 (mol·L^{-1})
1 m	46	0.50
2 m	22	0.29
3 m	13	0.10
5 m	6.1	0.04
7 m	2.8	0.02

Table S3. Mode assignments in the 300–600 cm^{-1} region based on the Raman spectrum of the 0.3 m electrolyte cell at OCV.

**Raman shift (cm^{-1})^{a}**	**Mode Assignment**	**References**
364 m	COC bending, CCO deformation, C–C stretching of the *tgt* conformation of DME	5
369 m	S–S stretching of S_8^{2-}	6-8
399 sh,vw	DME, unspecific mode of CO and CC vibrations	5
400 m	S–N bending of TFSI	9
400 m	S–S stretching of various S_n^{2-} (n = 4–8)	6-8
450 m	S–S stretching of various S_n^{2-} (n = 4–8)	6-8
468 w	S–S stretching of short chain PSs, mainly S_4^{2-}	6-8
511 sh	S–S stretching of S_8^{2-}	6-8
534 vs	S–S stretching of $S_3^{\bullet-}$	6-8
536 vw	S–C bending of TFSI	9
560 vw	SO_2 bending of TFSI	9
574 vw	CF_3 bending of TFSI	9
597 vw	SO_2 bending of TFSI	9

^{a} Notations on intensity: (v)s – (very)strong, sh – shoulder, m – medium, (v)w – (very)weak.

REFERENCES

(1) de la Cruz, C.; Molina, A.; Patil, N.; Ventosa, E.; Marcilla, R.; Mavrandonakis, A. New insights into phenazine-based organic redox flow batteries by using high-throughput DFT modelling. *Sustainable Energy Fuels* **2020**, *4* (11), 5513-5521, 10.1039/D0SE00687D.

(2) *CRC Handbook of Chemistry and Physics*; CRC Press/Taylor and Francis, Boca Raton, FL, Internet Version 2010.

(3) Wohlfarth, C. *Surface Tension of Pure Liquids and Binary Liquid Mixtures*; Springer Berlin, Heidelberg, 2017.

(4) Kottarathil, A.; Slim, Z.; Ahmad Ishfaq, H.; Jeschke, S.; Żukowska, G. Z.; Marczewski, M.; Lech, K.; Johansson, P.; Wieczorek, W. The Role of the Anion in Concentrated Electrolytes for Lithium-Sulfur Batteries. *J. Electrochem. Soc.* **2024**, *171* (7), 070506.

(5) Yoshida, H.; Matsuura, H. Density Functional Study of the Conformations and Vibrations of 1,2-Dimethoxyethane. *J. Phys. Chem. A* **1998**, *102* (16), 2691-2699.

(6) Bouchal, R.; Boulaoued, A.; Johansson, P. Monitoring Polysulfide Solubility and Diffusion in Fluorinated Ether-Based Electrolytes by Operando Raman Spectroscopy. *Batteries & Supercaps* **2020**, *3* (5), 397-401.

(7) Hannauer, J.; Scheers, J.; Fullenwarth, J.; Fraisse, B.; Stievano, L.; Johansson, P. The Quest for Polysulfides in Lithium–Sulfur Battery Electrolytes: An Operando Confocal Raman Spectroscopy Study. *ChemPhysChem* **2015**, *16* (13), 2755-2759.

(8) Hagen, M.; Schiffels, P.; Hammer, M.; Dörfler, S.; Tübke, J.; Hoffmann, M. J.; Althues, H.; Kaskel, S. In-Situ Raman Investigation of Polysulfide Formation in Li-S Cells. *J. Electrochem. Soc.* **2013**, *160* (8), A1205.

(9) Rey, I.; Johansson, P.; Lindgren, J.; Lassègues, J. C.; Grondin, J.; Servant, L. Spectroscopic and Theoretical Study of (CF3SO2)2N- (TFSI-) and (CF3SO2)2NH (HTFSI). *J. Phys. Chem. A* **1998**, *102* (19), 3249-3258.
